# Manipulation of Biological Cells Using a Robot-Aided Optical Tweezers System

**DOI:** 10.3390/mi9050245

**Published:** 2018-05-17

**Authors:** Mingyang Xie, Adnan Shakoor, Changcheng Wu

**Affiliations:** 1 College of Automation Engineering, Nanjing University of Aeronautics & Astronautics, Nanjing 211106, China; changchengwu@nuaa.edu.cn; 2 Department of Mechanical and Biomedical Engineering, City University of Hong Kong, Hong Kong, China; ashakoor2-c@my.cityu.edu.hk

**Keywords:** optical tweezers, cell manipulation, autonomous manipulation, micro/nano robot

## Abstract

This article reviews the autonomous manipulation strategies of biological cells utilizing optical tweezers, mainly including optical direct and indirect manipulation strategies. The typical and latest achievements in the optical manipulation of cells are presented, and the existing challenges for autonomous optical manipulation of biological cells are also introduced. Moreover, the integrations of optical tweezers with other manipulation tools are presented, which broadens the applications of optical tweezers in the biomedical manipulation areas and will also foster new developments in cell-based physiology and pathology studies, such as cell migration, single cell surgery, and preimplantation genetic diagnosis (PGD).

## 1. Introduction

Optical tweezers (OTs) are scientific instruments that utilize a highly focused laser beam to exert a trapping force and torque onto microscopic particles where the trapping forces are in the order of piconewtons [1,2]. OTs function as a special robot end-effector to trap and manipulate microparticles ranging from tens of nanometers to tens of micrometers. Due to the advantages of precision, flexibility, and noninvasive manipulation of microparticles, OTs have been widely utilized in a variety of biomedical research and clinical applications [3,4,5] including cell transportation [6], cell reorientation [7], cell sorting [8], cell fusion [9], cell stretching [10], cell assembly [11], and characterization of the mechanical properties of biological cells [12], etc.

**Figure 1:**
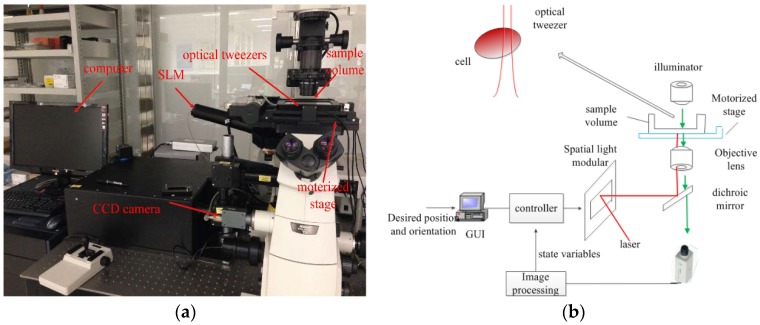
Robot-aided holographic optical tweezers cell manipulation system. (**a**) Experimental setup; and (**b**) illustration of the light path diagram.

With ongoing development trends towards cell manipulation with high precision, complexity, and flexibility, developing an autonomous cell manipulation framework is urgently needed. Fortunately, various cell manipulation strategies utilizing robot-tweezers have been developed over the past few decades. A typical robot-aided OTs manipulation system consists of an executive, a sensory, and a control module. The executive module is implemented by the optical trapping force, the sensory module consists of a charge-coupled device (CCD) camera and a microscope, while the control module adjusts the focal position of the optical trap. Combined with holographic technology, the focused laser beam can be split by a spatial light modular (SLM) into multiple optical traps simultaneously, and the focal position of each optical trap is well controlled independently in 3D. Therefore, a large number of biological cells can be manipulated simultaneously utilizing holographic optical tweezers (HOT). Figure 1 illustrates a robotically-controlled cell manipulation system equipped with HOT.

Autonomous cell manipulation that incorporates with the techniques of robotics, automation, microelectromechanical systems (MEMS), can achieve cell manipulation with high precision, robustness, and reliability, which is vitally important to many cell-based engineering applications, such as single cell surgery, PGD, targeted therapy, etc. The challenges of autonomous cell manipulation utilizing OTs present in sensory, planning, control, and design of end-effectors. Due to different dynamics such as the controlled objects, operation environment, disturbance, etc., the methodologies to address these challenges in micro/nano robotics are different to the methods for macroscale robotics. For instance, the sensory algorithms need to distinguish and identify the particles of interest (such as biological cells, grasping particles) from the other irrelevant particles, and these different types of particles are always in various sizes; the planning methods need to incorporate fluid motion, viscous drag force, and speed constraint of OTs into consideration, etc.; the control algorithms deal with model and unmodeled uncertainties, external disturbance (such as Brownian motion, vibration), stable optical trapping of biological cells, and multi-OTs cooperative control. Moreover, the optimal design of the end-effector configuration is also required when manipulating soft cells, large cells, opaque particles, and the laser-sensitive cells.

This article reviews the typical autonomous frameworks of the optical manipulation of biological cells, consisting of direct and indirect manipulation strategies. The latest achievements in the aforementioned cell manipulation strategies are also covered, which mainly focus on multi-OTs cell manipulation, multi-DOF (degree of freedom) cell orientation control, and in vivo cell manipulation. Furthermore, the integrations of OTs with other micromanipulation tools, such as microfluidic chips, microneedles, and MEMS sensors, have also been reviewed. These integrations expand the manipulation abilities with high complexity, throughput, and reliability, and will contribute to cell-based physiological and pathological studies, such as single cell surgery, PGD, target therapy, etc. The existing challenges and research trends of the aforementioned autonomous optical manipulation of biological cells are also presented.

## 2. Cell Manipulation Strategies

This section reviews direct and indirect cell manipulation strategies using OTs. The latest achievements in direct manipulation of biological cells will be introduced, mainly covering multi-DOF cell orientation control, and in vivo cell manipulation while, for indirect manipulation, the progress focuses on employing grasping formation and pushing-based strategies to achieve autonomous cell indirect manipulation, and the grasping formation functions as the end-effector (denoted as gripper) were manipulated by multi-OTs.

### 2.1. Manipulation Using Direct Optical Trapping

Direct optical trapping of cells is the simplest manipulation strategy. Numerous autonomous cell manipulation frameworks using a robot-aided optical tweezers system have been developed over the past decade [13,14,15,16]. The dynamics of the trapped cell was modelled after analysis and calibration of the exerted forces onto the cell [13,17],

(1) $$m\ddot{q}=F_{trap}-F_{drag}$$

(2) $$F_{trap}=\{\begin{array}{c} k_{1}(l-q),\;0<l-q<r_{0}\\ -k_{2}(l-q)+c,\;l-q\text{>}r_{0} \end{array}$$

(3) $$F_{drag}=\beta \dot{q}$$

where *m* is the mass of the trapped cell; $q\in {ℜ}^{3}$ denotes the position coordinates of the center of the cell 0; *l* is the position coordinates of the center of the optical trap; $k_{1}$ and $k_{2}$ are the trapping stiffness before and after the critical displacement $r_{0}$, respectively; *β* represents the viscous coefficient. Note that the optical trapping force increases as the offset increases when the offset between the optical trap and cell *l* − *q* is smaller than, and the optical trapping force decreases when the offset *l* − *q* exceeds to the critical displacement $r_{0}$, and becomes to zero when the cell is completely outside the optical trap. Therefore, the offset between the optical trap and the cell *l* − *q* should be well confined within the critical distance $r_{0}$ for stable and reliable optical manipulation of biological cells.

**Figure 2:**
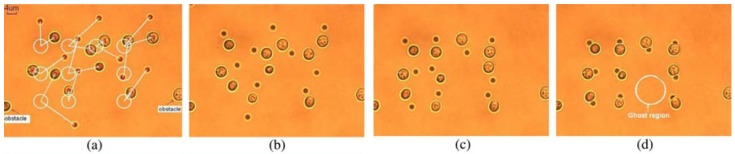
Snapshots of moving two groups of microparticles into arrays with an artificial potential field-based controller. (**a**) 0 s; (**b**) 1 s; (**c**) 3.5 s; (**d**) 4.5 s. Reproduced with permission from [20].

**Figure 3:**
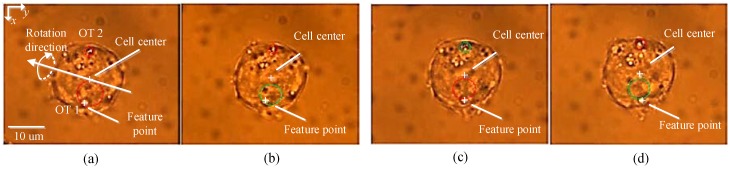
Snapshots of cell out-of-plane rotation control using two optical traps. (**a**) 0 s; (**b**) 0.25 s; (**c**) 2 s; (**d**) 5 s. Reproduced with permission from [35].

**Figure 4:**
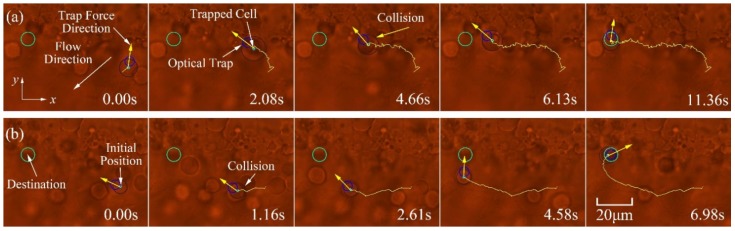
Snapshots of in vivo red blood cells (RBCs) transportation in a living zebrafish; (**a**) disturbance compensation controller; and (**b**) P-type controller. Reproduced with permission from [37].

Based on the dynamic model formulated in Equations (1)–(3), a synchronous control strategy was developed for single cell position control as well as multiple cells [18]. In order to avoid collisions with other cells or obstacles, a path planning approach based on a rapidly exploring random trees (RRT) algorithm was present for the achievement of cell transportation in a dynamic micro-environment [19]. Artificial potential field-based control frameworks were also developed to move large numbers of cells into a desired topology while avoiding collisions [20,21,22,23], as demonstrated in Figure 2. Furthermore, some control strategies were introduced to keep the trapped biological cells within the critical displacement of the optical trap for the achievement of stable cell manipulation [24,25]. A dynamic trapping and manipulation framework for cells was introduced in [26], which addressed the problem of cell trapping and manipulation using one controller, avoiding control strategies switching from one to another. A theoretical framework that integrated the interaction between the manipulator of the laser source and the trapping cell into consideration, while most of the reported cell manipulation frameworks adopted open-loop strategies to control the position of the laser focus. A trajectory tracking controller was developed for the optical manipulation of biological cells using observer techniques, where the calibration of the Jacobian matrix from the Cartesian space to the image space of the CCD camera and the measurement of the velocity of cell are not required [27]. Robust control frameworks were also developed to address the problems of dynamic model parameters uncertainties and limited field of view [28,29]. Additionally, to avoid the requirement of high-order state variables, which are difficult to measure, a simple PD control architecture was introduced to achieve cell position regulation, which also takes the interaction between the manipulator of the laser source and the trapped cell into consideration [30]. Moreover, to improve cell transportation speed and efficiency, a switching control approach was developed to achieve high transportation speed while maintaining stable optical trapping of biological cells [31], where a switching geometrical model that takes cell trapping, stable optical trapping of cells with high speed, and obstacle avoidance into consideration. Recently, much progress has been achieved in cell orientation control and in vivo cell transportation control. A general dynamic model, which also takes cell rotation into consideration, has been developed [32]. By using a T-matrix approach for computational modelling OTs [33], the relationship between the applied torques onto the cell and the position coordinates of the OTs within the trapped cell is characterized. Furthermore, a simplified dynamic model of multi-DOF cell rotational control, under the action of two optical traps, was derived. Based on the simplified dynamic model, cell rotational control in the microscope optical plane (referred to in-plane rotation) has been realized [34], and cell rotational control out of the microscope optical plane (referred to out-of-plane rotation) was reported in [35], as shown in Figure 3. In vivo manipulation of biological cells have recently attracted considerable attention due to their extensive applications in precision medicine, such as drug delivery, in vivo cancer targeted therapy, and in vivo manipulation of microrobots, etc. A methodology was presented in [36] to calibrate the optical trapping stiffness in vivo by measuring the flow profiles and drag forces imposed to the optical trapped cell, which contributes to assessing biomechanics in vivo. An in vivo cell transportation control framework was established in [37], and a disturbance compensation strategy was presented to overcome the complex in vivo environmental influences, as illustrated in Figure 4. Furthermore, to avoid collisions when performing in vivo cell transportation, an automated control approach integrated with obstacle avoidance function was developed for in vivo cell transportation control, where a collision-avoidance vector method was introduced to avoid obstacles during the target cell transportation [38].

Banerjee et al., developed a stochastic dynamic programming based on motion planning framework to move the trapped particles while avoiding collisions with random moving particles, and the proposed motion planning framework adopted a modifying version of an infinite-horizon partially-observable Markov decision process algorithm where the form of the payoff function was changed and introduced a *t* variable into the convergence loop [39].

Direct optical trapping of cell manipulation is simple and fast, however, the disadvantages of this type of cell manipulation are obvious, on one hand, the reported cell manipulation strategies easily cause photo-damage to the trapped biological cells due to direct laser exposure; on the other hand, the types of cell manipulation is single which cannot meet many complex applications. With the trend toward complex cell manipulation, developing an autonomous framework that can perform various types of cell manipulation is urgent needed. Moreover, robust sensory and control strategies are also required to address when performing in vivo cell manipulation within a complex environment, such as fluid motion, dynamic model uncertainties, and external disturbances.

### 2.2. Indirect Manipulation

As mentioned previously, the direct optical trapping strategies are not suitable for manipulating laser-sensitive biological cells due to the potential photo-damage. To avoid direct laser exposure, many indirect-based cell manipulation strategies have been developed recently, and these strategies can be divided into three categories denoted as gripper formation, pushing-based, and inert particle attachment.

#### 2.2.1. Gripper Formation

For trapping and manipulating a target biological cell, several dielectric beads (such as polystyrene beads, silica beads) are individually trapped by OTs and driven to form a desired topology around the target cell, thus the trapped microbeads function as special end-effectors to trap and manipulate the target cell to the desired location in an indirect manner, and this type of indirect cell manipulation strategy can reduce 90% laser exposure.

**Figure 5:**
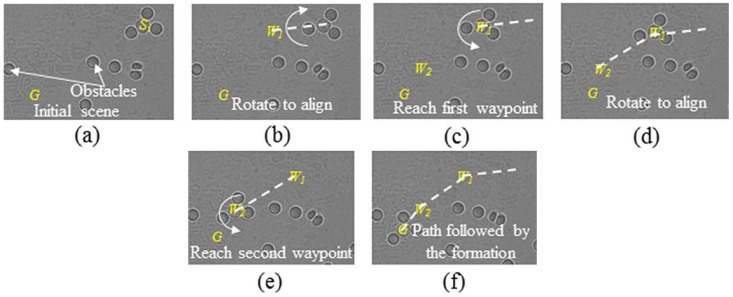
Transportation of a bead utilizing a three-bead gripper formation. (**a**) The initial state of the gripper *S_i_*; (**b**) rotating maneuver; (**c**) translating maneuver to reach the waypoint *W*_1_; (**d**) rotating maneuver to align it towards the waypoint *W*_2_; (**e**) translating maneuver to reach the waypoint *W*_2_; and (**f**) the target bead reached the desired position using a series of translating and rotating maneuvers. Reproduced with permission from [40].

**Figure 6:**
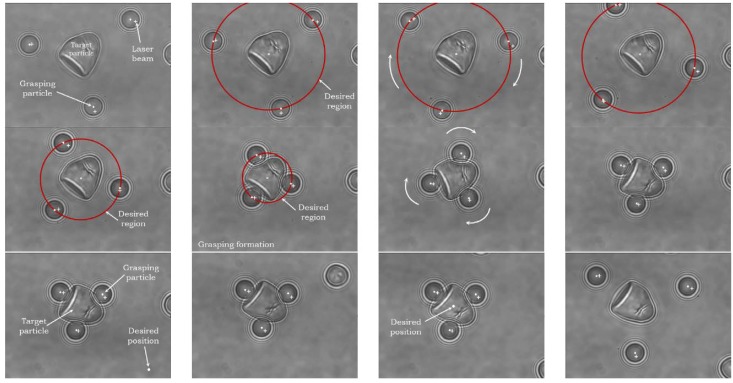
Snapshots of gripping and manipulation of a bell-like target particle. Reproduced with permission from [41].

Chowdhury et al. developed a control and planning approach for indirect cell manipulation utilizing silica beads as a gripper formation [40], as demonstrated in Figure 5. A collision-free path for the gripper formation was generated by utilizing an A*-based path planning algorithm, and a designed cost function was introduced into the planner to minimize the transportation time, moreover, a feedback controller was formulated to ensure the manipulated cell tracking the trajectory using a series of predefined maneuvers, including translating, rotating, and retaining. However, the dynamic interactions between the target cell and the gripper beads, and the stability analysis of the feedback controller were not taken into consideration. Meanwhile, the proposed method only evaluated by transporting spherical cells. To address these challenges, Cheah et al. presented a grasping and manipulation strategy of biological cells using robotically controlled multiple optical traps [41]. Several latex micro beads were independently trapped by OTs to form a gripper, and then a region control strategy was developed to manipulate the trapped latex beads to form the desired gripper topology. By considering the interactions among the target cell, gripping beads, and robotic manipulator, an integrated dynamic model was established and then a sliding controller was derived to achieve cell position and orientation control in 2D, the proposed approach can also be applied to manipulate cells with irregular shape, as illustrated in Figure 6. The research trend for gripper formation-based indirect cell manipulation is to develop a framework to synchronously realize cell position and orientation control in 3D, where the challenges existing in gripper formation design, dynamic modelling, cell state variable (position, orientation) extraction in 3D, etc.

#### 2.2.2. Pushing-Based

The gripper formation-based cell manipulation strategy can reduce 90% of the laser power from irradiating onto the cell; however, the physiological characteristics of the light-sensitive cells were still affected due to the extra 10% laser exposure using gripper formation. To address this problem, Thakur et al. proposed a pushing-based cell manipulation strategy, where an optically-trapped bead pushed an intermediate bead that in turn pushed the target cell towards its desired position, therefore, the trapped bead acted as the actuator and the intermediate bead served as the end-effector [5]. The dynamic-based simulation model of the indirect pushing manipulation was proposed, and a feedback planner including three maneuvers, namely, push, align, and backup, was proposed. The planner could deal with measurement uncertainties, and the parameters of the planner were tuned based on genetic algorithm, both of which can increase the robustness of the pushing-based manipulation strategy. The proposed pushing-based cell manipulation approach only consider two beads, which cannot be applied to transport cells with irregular shapes. Furthermore, this group improved the indirect pushing-based method by using two actuator beads and an intermediate bead, and the improved method was applied to transport and rotate a dynamic *Dictyostelium discoideum* cell with an irregular shape in 2D [42]. These pushing-based cell manipulation strategies have the following disadvantages: first, the developed approaches did not consider complex conditions such as sensing uncertainty, fluid viscosity, laser power; second, the stability analysis of the proposed closed-loop frameworks were not presented; third, achieving cell position and orientation control in 3D utilizing pushing-based manipulation strategy is still a challenge.

#### 2.2.3. Inert Particle Attachment

**Figure 7:**
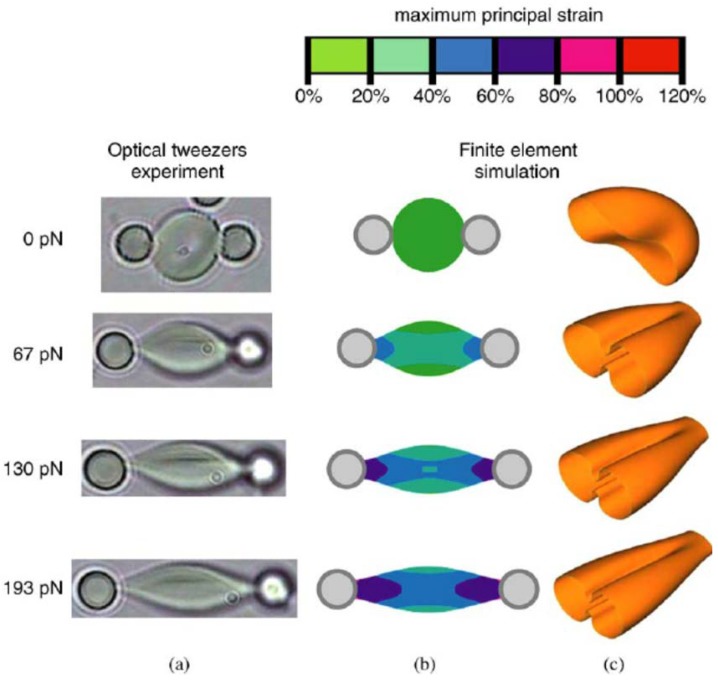
Snapshots of RBCs stretching using attached polystyrene beads; (**a**) experimental method to calibrate the relationship between the cell stretching force and the corresponding deformation of human red blood cells (RBCs); (**b**,**c**) mechanical model to establish the relationship between the force and deformation using finite element analysis.

The mechanical properties of the biological cells are relevant to their physiological and pathological characteristics, and the physiological status of the target cell can be reflected through the calibration of mechanical parameters of the target cell such as shear moduli, Young’s modulus, and stiffness, which involves cell pulling manipulation. The aforementioned gripper formation and pushing-based cell manipulation strategies cannot perform cell pulling manipulation. To solve this problem, inert particle attachment-based cell manipulation was developed where the target cell attached to inert particles using adhesive. By stretching the optically-trapped inert particles, the cell of interest could be stretched or pulled indirectly. Tan et al. experimentally established the relationship between the cell stretching force and the corresponding deformation of human red blood cells (RBCs) under different osmotic conditions [43]. The streptavidin-coated polystyrene beads were attached to RBCs under incubation at 25 °C for one hour. Meanwhile, a mechanical model of the stretched cell was developed utilizing finite element analysis. Comparing the experimental data to the model results, the shear moduli of RBCs under different osmotic conditions was characterized. These results demonstrated that osmotic pressure affected the mechanical properties of biological cells, and will provide insight into the relationship between the mechanical and physiological properties of biological cells. Figure 7 demonstrates the procedures of RBCs stretching using attached polystyrene beads. Similar studies to measure the mechanical properties of RBCs can be found in [44].

Maruyama et al. developed a gel-based microtool for on-chip cell manipulation [45]. The gel microtools were made of hydrophilic photo-crosslinkable resin, and the attachment between the cell and the gel microtools were functionalized by spiropyran chromospheres, which are a photochromic polymer. The adhesiveness was realized by immersing into electrolyte solution after ultraviolet (UV) illumination, and the cell was detached from the microtools after visible light irradiation. By adjusting the concentration of the electrolyte solution, the adhesiveness could be well-controlled. The developed approach can be applied to perform cell pulling as well as pushing manipulation. By coating the microtools with a pH indicator, the pH value of the cell can be characterized by identifying the color of the microtools. This group also developed a massive parallel self-assembly technique to produce an arbitrary shape of the microtools [46]. The microparticles, made of polystyrene, were dispersed into a silicon substrate with microtool patterns, and autonomously aggregated by surface tension. The aggregated particles were fused to form the desired pattern by heating above the glass transition temperature. Compared with the conventional photo fabrication of microtools, the proposed method exhibited a higher trapping efficiency of the microtools and massive production of microtools with an arbitrary shape.

This type of cell manipulation strategy exhibits the following disadvantages. On one hand, the physiological characteristics of cells may be altered due to using protein adhesive, the attached beads are also difficult to release from the target cell after manipulation; on the other hand, the manipulation precision is low and the biological samples are easily contaminated due to using an open-loop manipulation strategy.

## 3. New Routes in Integrated Platform

The integration of OTs with other manipulation tools, such as microfluidic chips, microneedles, and MEMS, enhance the manipulation capabilities to perform complex biological experiments, which takes advantage of the complementary merits provided by these manipulation tools.

Chowdhury et al. developed an OTs-aided microfluidic chamber to achieve large numbers of cell transportation. A Langevin equation was developed to simulate cell motion within the chamber by considering fluid forces. The probabilities of the cell reaching the outlets of the chamber from different locations within the microfluidic chamber can be predicted with the developed simulator. A planner was then developed to generate collision-free paths, which utilized fluid flow together with the offline data determining the releasing locations of the cells. The proposed framework realized massive cell transportation while reducing the potential photo-damage [47]. In addition, Wang et al. developed a sorter to realize small cell population sorting by utilizing optical tweezers integrated with microfluidic chip [8]. Image processing algorithm was developed to identify the target cells, and then multiple optical traps were generated to manipulate the target cells to the collector with high accuracy, purity, and recovery rate.

**Figure 8:**
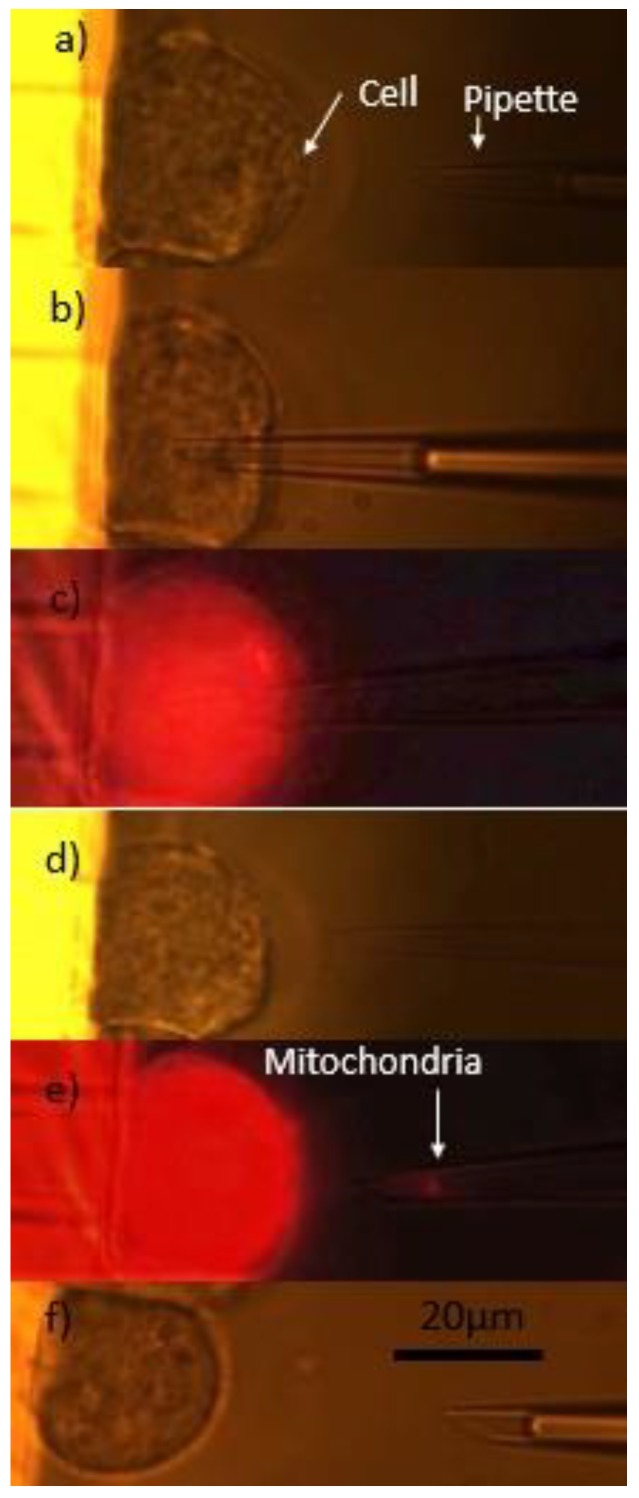
Snapshots of extraction of cell mitochondria. (**a**) Detection of position coordinates of the biopsied mitochondria; (**b**) moving the micropipette to the mitochondria; (**c**) aspiration of mitochondria; (**d**,**e**) moving the micropipette out of the cell; and (**f**) releasing the mitochondria from the micropipette. Reproduced with permission from [48].

A platform that integrated optical tweezers and microneedle was developed to perform cell biopsy, which is an important technique to extract intracellular organization and/or components for disease diagnosis and treatment at the single cell-level [48]. The proposed system adopted OTs to perform cell position and orientation control, and after the target cell was placed at the desired posture, the integrated micropipette was manipulated to the biopsy site, then the dye-labeled mitochondria was extracted using an external injection pump, the detailed procedure of cell biopsy was illustrated in Figure 8. In addition, this group also introduced a methodology to measure cell protrusion force, which drives cell migration, using optical trapped polylactic-co-glycolic acid (PLGA) bead [49]. The trapped chemoattractant-loaded PLGA bead was placed near the target cell as a stimulator and force sensor, and the protrusion force drove the bead away from the OTs. The deviation between the PLGA bead and the OTs can be calculated using the image processing algorithm and was utilized to calibrate the optical trapping force where the trapping force equaled the cell protrusion force at equilibrium. Therefore, the OTs functioned as a sensor to measure cell protrusion force in this work. The presented research quantitatively characterized the mechanism of cell migration and will contribute to revealing the mechanism of cancer metastasis.

Moreover, the OTs were also integrated with a MEMS sensor to measure the singular cell mass with high accuracy [50], where the target cell was accurate positioned within the MEMS sensor for long-term, repeatable measurement. Researchers at Korea University developed a micro-electrode embedded microfluidic chip combined with optical tweezers to measure dielectrophoretic (DEP) force of RBC, and utilized the DEP characteristics to assess the physiological conditions of RBCs [51]. It is indicated that DEP forces can function a vital important parameter to assess whether the RBCs are fresh and not exposed to oxidative stress.

It is concluded that the biological cells can be manipulated and interrogated from extracellular to intracellular utilizing the integrated micro/nano-manipulation system, and multidimensional information about biological cells can be acquired, which will contribute to having an insight into the physiological and pathological mechanisms of intracellular activities. However, the integration degree of the reported platforms is still low, and limited physiological parameters were required. The research trends may focus on the optimal design and integration of various micro/nano-manipulation tools, cooperation, switching, and robust control of different modes in a complex environment.

## 4. Discussion and Conclusions

This paper reviewed the autonomous cell manipulation strategies utilizing OTs, and also introduced the integrated platforms to achieve more complex manipulation and measurement of biological cells at the subcellular level. It is concluded that the manipulation complexity increased from direct trapping to gripper formation/particle attachment to pushing-based manipulation, while the potential photo-damage significantly decreased accordingly. As reported in [4], the photo-damage can be totally eliminated for pushing-based cell manipulation, while the occurrence rates of photo-damage are 33% and 67% for gripper formation/particle attachment and direct trapping, respectively. Direct optical trapping of cell manipulation was the simplest and fastest manipulation strategy; however, it may cause photo-damage to the trapped biological cells and was not suitable for the manipulation of laser-sensitive cells. The particle attachment-based method can realize diverse cell manipulations, such as cell pulling, which plays a vital important role in the characterization of cell mechanical properties; however, the attached beads were difficult to release from the target cell in the presence of adhesive coatings, and this approach required a longer experiment preparation time in the orders of several minutes. The experimental preparation time, cell transportation time, laser radiation, and manipulation complexity of gripper formation-based cell manipulation were moderate. The transportation time and the manipulation complexity of the pushing-based method were the highest due to involving many types of maneuvers, while the potential photo-damage can be totally eliminated. The beads were easily released from the cell for both gripper formation and pushing-based cell manipulation when compared to particle attachment-based strategy, moreover, these two strategies were suitable for manipulating large cells with irregular shapes or opaque particles. Table 1 makes a comparison of these different types of cell manipulation strategies.

The vast majority of the reported cell manipulation studies remained at the extracellular level, and the manipulation strategy was sole, both of which cannot meet complex applications at the subcellular level, such as, single cell surgery. The development of an unified framework that can perform various cell manipulation strategies at subcellular level in 3D is urgently needed, which requires advancements in micro/nano robotics, including coupled planning and control algorithms, the switching and coordination of different control strategies, visual perception, and reconstruction in 3D, etc. With the trend towards cell manipulation at the subcellular level to characterize the cell physical and physiological properties and, furthermore, to insight into the mechanisms of intracellular activities, it is very necessary to integrate OTs with other platforms, such as optoelectronic, optomagnetic, optomechanical, and optofludic platforms, to perform cell manipulation with high complexity, diversity, and precision, which contributes to acquiring multidimensional information about biological cells to have an insight the physiological and pathological mechanisms of intracellular activities.

## Figures and Tables

**Table 1:** Comparison of cell manipulation strategies.

Manipulation Strategy	Direct Trapping	Gripper Formation	Pushing-Based	Particle Attachment
Manipulation complexity	Simplest	Moderate	Most complicated	Moderate
Photo-damage occurrence rate	67%	33%	No	33%
Manipulation types	Transportation, rotation	Transportation, rotation, pushing	Transportation, rotation, pushing	Transportation, rotation, pushing, pulling
Experimental preparation	A few minutes	A few minutes	A few minutes	Tens of minutes
Transportation time	Less than a minute	A few minutes	Tens of minutes	A few minutes
Cell release	Easy	Easy	Easy	Very Difficult
